# Production and Characterization of β-Cyclodextrin Inclusion Complexes Containing Conjugated Linoleic Acid from Njangsa (*Ricinodendron heudelotii*) Seed Oil

**DOI:** 10.3390/molecules31193560

**Published:** 2026-10-07

**Authors:** Anh T. L. Nguyen, Cecile T. Ojong, Pierre Dutilleul, Alberta N. A. Aryee

**Affiliations:** 1 Food Science and Biotechnology Program, Department Human Ecology, College of Agriculture, Science and Technology, Delaware State University, 1200 N DuPont Highway, Dover, DE 19901, USA; 2 Department of Plant Science, Agricultural and Environmental Sciences, McGill University, 21111 Lakeshore Road, Sainte-Anne-de-Bellevue, QC H9X 3V9, Canada

**Keywords:** alkali isomerization, conjugated linoleic acid, β-cyclodextrin, complexation, oxidative stability

## Abstract

Njangsa seed oil (NSO) is rich in polyunsaturated fatty acids and represents an underexplored plant source for producing conjugated linoleic acid (CLA). This study explored the production of CLA from NSO via alkali isomerization, as well as and its stabilization by β-cyclodextrin (β-CD) inclusion complexation. A 3-factor factorial design was used to optimize reaction conditions, yielding a maximum purified CLA content of 46.42% from 2.5 g NSO at 180 °C for 3 h, with cis-9, trans-11 CLA as the predominant isomer. Inclusion complexation produced microparticles averaging 19.73 µm with 83.36% inclusion efficiency. Complexed CLA showed lower peroxide, para-anisidine, and thiobarbituric acid values than free CLA, indicating improved oxidative stability. Fourier transform infrared spectroscopy showed spectral changes consistent with interactions between the CLA-rich fraction and β-CD, while scanning electron microscopy and X-ray diffraction revealed changes in morphology and crystalline organization, respectively. These findings demonstrate the feasibility of producing NSO-enriched CLA and enhancing its oxidative stability through β-CD inclusion complexation.

## 1. Introduction

Conjugated linoleic acid (CLA) comprises positional and geometric isomers of linoleic acid and occurs naturally in ruminant-derived foods, particularly meat and dairy products, where it is formed largely through ruminal biohydrogenation [1,2]. Linoleic acid-rich plant oils have also gained attention as substrates for CLA production through chemical and biological conversion [3]. Recent studies have reported metabolic effects of CLA in animal and human models. In young horses, CLA supplementation was associated with skeletal muscle mitochondrial adaptations, although clear improvements in the measured antioxidant and oxidative-stress markers were not observed [4]. In obese mice, 10,12 CLA increased HDL particle concentration, enhanced HDL anti-inflammatory activity, and increased cholesterol efflux capacity [5]. In a high-fat diet-induced insulin resistance mouse model, CLA attenuated hyperglycemia, hyperlipidemia, glucose intolerance, and insulin resistance while reducing inflammation and enhancing fatty acid oxidation and insulin signaling [6]. More recently, a randomized controlled trial in adults with high body fat showed that 12 weeks of CLA supplementation suppressed de novo lipogenesis and was associated with changes in lipid-related metabolic pathways [7]. Emerging evidence also links CLA to cancer-related mechanisms, including peroxisome proliferator-activated receptor gamma (PPAR-γ)-mediated protective responses in colorectal cancer models [8]. Growing interest in CLA has spurred the development of various strategies aimed at enhancing its production. Dietary approaches, including supplementation with oilseeds rich in linoleic and linolenic acid, have been employed to modify the fatty acid composition of milk and increase its CLA content [2,9,10]. Beyond dietary enrichment, linoleic acid-rich plant oils can serve as substrates for CLA production. For instance, Rezavand Hesari et al. [3] demonstrated the bioconversion of grapeseed oil to cis-9, trans-11 and trans-10, cis-12 CLA using *Bacillus coagulans*. Chemical isomerization provides another route for CLA enrichment, with Ramírez-Brewer et al. [11] demonstrating efficient alkaline-mediated conversion of linoleic acid in sesame oil to CLA. CLA production is influenced by the conversion system and processing variables, including substrate composition, temperature, pH, reaction or incubation time, and the chemical or microbial conditions [3,11].

Despite its biological relevance, CLA is susceptible to oxidative degradation, which can limit its application. The oxidative stability of CLA is influenced by its molecular form and storage conditions [12,13]. To address this limitation, methods such as encapsulation and complexation have been employed effectively to protect oxidation-sensitive lipids and facilitate their incorporation into food products [14]. More recently, whey protein-based delivery systems have shown potential for stabilizing CLA; Li et al. [15] reported lower primary and secondary oxidation products in CLA emulsions stabilized with glycosylated whey protein hydrolysates compared with emulsions stabilized with unmodified whey protein isolate. Among the different carriers, cyclodextrins (CDs) have received considerable attention for the delivery of hydrophobic and poorly water-soluble compounds. CDs are cyclic oligosaccharides composed of α-(1→4)-linked D-glucopyranose units and occur primarily as α-, β-, and γ-CD, which contain six, seven, and eight glucose units, respectively [16,17]. Their truncated-cone structure provides a hydrophilic outer surface and a relatively hydrophobic internal cavity, allowing hydrophobic guest molecules to form inclusion complexes within the cavity [16,17,18,19]. This structural characteristic has supported the use of CDs in food and other delivery applications. Recent studies demonstrate the continued application of β-CD for the delivery and stabilization of oxidation-sensitive lipids and bioactive compounds. Ghorbanzade et al. [20] complexed fish oil with β-CD and reported improved oxidative stability following incorporation into yogurt, while Li et al. [21] demonstrated successful inclusion complexation of garlic oil with β-CD, resulting in improved stability and controlled-release properties. More recently, Huang et al. [22] investigated the inclusion complexation of α-linolenic acid ethyl ester from flaxseed oil with β-CD, further demonstrating the applicability of β-CD to highly unsaturated fatty acids. Current approaches have also extended to modified CDs; for instance, Sun et al. [23] reported that 2-hydroxypropyl-β-cyclodextrin complexation improved the aqueous solubility and stability of the lipophilic carotenoid fucoxanthin. These developments underscore the on-going use of native β-CD as an inclusion matrix, while also highlighting the potential of modified CDs for the delivery lipids and bioactives.

This presents a promising avenue for Njangsa seed oil (NSO), a relatively underexplored seed kernel oil from Njangsa (*Ricinodendron heudelotii* (Baill.) Heckel. Notably NSO is abundant in polyunsaturated fatty acids (PUFAs), including linoleic acid and α-eleostearic acid [13]. Njangsa belongs to the Euphorbiaceae family and occurs across tropical Africa, including Cameroon and Côte d’Ivoire [24,25]. Traditionally, the edible seed kernels are crushed and used as a thickener and spice in sauces, soups, and other dishes due to their distinct flavor [24,26]. Beyond culinary uses, NSO has been investigated for pharmaceutical and industrial applications, due to its bioactive properties and potential use as a modifier in alkyd resins and surface-coating materials [13,24,25]. Diversifying the markets for its seed kernels, proteins, and oils could support greater value addition and provide economic benefits to local communities and emerging markets [24,25]. Although techniques such as alkali isomerization, urea-assisted enrichment, and β-CD inclusion complexation are well-established, their integrated application to produce, enrich, and stabilize a CLA-rich fraction from NSO remains underexplored. The CLA-β-CD formulation was therefore developed as a proof-of-concept approach to improve the oxidative stability of CLA derived from NSO, with potential application as a stabilized ingredient in functional foods. Accordingly, the effects of reaction temperature, oil amount, and reaction time on CLA production from NSO were investigated. The condition yielding the highest CLA content was selected for further enrichment by urea-assisted fractionation, after which the purified CLA-rich fraction was complexed with β-CD and evaluated for inclusion efficiency and oxidative stability. Particle size distribution, morphology, molecular interactions, and crystalline characteristics were further analyzed using Fourier transform infrared (FTIR) spectroscopy, X-ray diffraction (XRD), and scanning electron microscopy (SEM).

## 2. Materials and Methods

### 2.1. Materials

Njangsa seed were purchased from a local market in Dover, DE, USA. Fatty acid methyl esters (FAMEs) reference standard 15A was obtained from Nu-Check Prep (Elysian, MN, USA). All other reagents including linoleic acid, conjugated methyl ester standard (O5632) were purchased from Sigma-Aldrich Chemical Co. (St. Louis, MO, USA), Fisher Scientific (Waltham, MA, USA), and The Lab Depot Co. (Dawsonville, GA, USA).

### 2.2. Methods

#### 2.2.1. Solvent Extraction of Njangsa Seed Oil

Njangsa seeds were tempered in an oven at 25 °C, pulverized in a coffee grinder, and stored in airtight bags at −20 °C until needed. One part of the ground seeds was twice extracted with two parts of hexane by stirring at 400 rpm for 1 h. The hexane supernatant was filtered using Whatman paper #1 and passed through anhydrous sodium sulfate (Na_2_SO_4_) to remove residual moisture. Hexane was removed from the filtrate using a rotary evaporator (Rotavapor R-3, BÜCHI, New Castle, DE, USA) under vacuum. The oil obtained (NSO) was stored in an amber bottle at −20 °C until needed.

#### 2.2.2. Preparation and Crystallization of CLA

CLA was prepared from NSO using glycerol and potassium hydroxide (KOH) according to the method described [27]. The effects of reaction temperature, amount of oil, and reaction time on the alkali isomerization to CLA were investigated using a three-way factorial design with three levels per factor; at temperature (150, 170 and 180 °C), amount of oil (2.5, 5.0, and 7.5 g) and reaction time (1, 3 and 6 h), with CLA (%) as the dependent variable or variable to maximize. Each of the 27 combinations of values of the three input variables was replicated three times. The experimental levels for the factors were selected based on preliminary experiments aimed at identifying suitable conditions for producing CLA from NSO. NSO, in amounts of 2.5, 5.0 or 7.5 g was added to a mixture of 15 mL glycerol and 5 g KOH. This mixture was then heated under reflux with magnetic stirring at 150, 170, and 180 °C. Aliquots of ~130 mg of the mixture were taken at 1, 3, and 6 h. The samples were cooled to room temperature (22–25 °C) and acidified with 1 mL 1 M HCl. Subsequently, 10 mL hexane and 5 mL 80% (*v*/*v*) methanol were added to the acidified samples and vortexed. After centrifuging (Heraeus Megafuge 8 centrifuge Waltham, MA, USA) the mixture, the upper layer was dried using anhydrous Na_2_SO_4_ and the hexane layer was removed using nitrogen and labeled NSO-CLA (CLA obtained from NSO). NSO-CLA was crystallized as described [27] using CLA urea weight ratios of 1:2, 1:3, 1:4, 1:5 or 1:6 (*w*/*w*). A suitable amount of urea was added to 1 g of NSO-CLA and 15 mL pre-warmed methanol. The mixture was incubated for 1 h at 60 °C with shaking in a water bath (Amerex Instruments, Concord, CA, USA). Subsequently the reaction mixture was allowed to crystallize in a refrigerator at 4 °C for 18 h. Following this, the urea complex (UC) fraction (the needle crystal) was filtered on Whatman paper #1 and separated from the mother liquor (ML). The ML fraction was transferred to a separatory funnel to which distilled water was added, and the mixture was acidified with 1 M HCl to pH < 2. Each 1 g CLA was extracted with 50 mL hexane and transferred to a clean separatory funnel. The hexane fraction was further washed with 30% (*v*/*v*) methanol, dried over anhydrous Na_2_SO_4,_ and removed using a Rotavapor R-3 rotary evaporator (BÜCHI, New Castle, DE, USA) and labelled filtered CLA fraction. The UC fraction was dissolved in warm 70% (*v*/*v*) methanol. The mixture was then transferred to a separatory funnel to which drops of 10 M sulfuric acid was added to reduce pH to <2. CLA was extracted with hexane and transferred to another clean separatory funnel. The hexane fraction was further washed with 30% (*v*/*v*) methanol, dried over anhydrous Na_2_SO_4,_ and removed using a rotary evaporator and labelled purified CLA fraction (NSO-CLA). Both fractions (filtered and purified CLA) were stored for further analysis.

#### 2.2.3. Fatty Acid Profile Analysis

The native NSO, along with the isomerized, filtered, and purified CLA fractions, were converted to FAMEs in accordance with the AOAC Method 996.01 and analyzed using gas chromatography (GC) (GC2010-plus, Shimadzu Corp., Columbia, MD, USA) equipped with a ZB-FAME, 30 m fused silica capillary column (0.25 mm internal diameter, 0.2 um film thickness; Phenomenex, Torrance, CA, USA) and coupled to a flame ionization detector (FID). Run conditions were: carrier gas and hydrogen; linear velocity at 40.0 mL/min; injector temperature at 260 °C; oven program at an initial temperature of 100 °C, hold for 2 min, then ramp up temperature at a rate of 10 °C/min to 140 °C, then ramp at a rate of 3 °C/min to 190 °C, and finally increase to 260 °C, at rate of 30 °C/min held for 2 min (total run time 30 min); and detector temperature at 240 °C. The reference standard mixture (15A) was used to confirm both the retention times and response factors for the GC-FID. Fatty acid composition was expressed as a percent of total identified fatty acids. FAMEs of CLA isomers were determined using an Intuvo GC system 5977B MSD (Agilent Technologies, Santa Clara, CA, USA) equipped with a fused silica DB FastFAME column (20 m × 0.180 mm, 0.20 µm particle size; Agilent Technologies). CLA isomers were identified by comparison of retention times with an authentic standard mixture (O5632).

#### 2.2.4. Formation of NSO-CLA-β-Cyclodextrin Inclusion Complexes

The purified CLA fraction (NSO-CLA) was complexed with β-CD using the co-precipitation method [28]. Each 5 g β-CD was dissolved in 50 mL co-solvent of ethanol and water (1:2 *v*/*v*) and magnetically stirred at 55 °C on a hot plate stirrer. CLA (1 g) was dissolved in warm 10% ethanol (*v*/*v*) and slowly added to 36, 45, 54, and 63 g of a β-CD–ethanol–water mixture. These formulation levels were selected to systematically evaluate the effect of increasing the β-CD-containing phase on complexation while maintaining the amount of CLA constant, as the guest-to-β-CD proportion can influence inclusion efficiency [22,28]. The mixtures were stirred at 55 °C for 4 h while the temperature decreased to room temperature. The final mixture was maintained at 4 °C for 12 h, followed by centrifugation at 1200 rpm for 10 min (Heraeus Megafuge 8 Centrifuge, Waltham, MA, USA). The uncomplexed oil (which floated on the top of the mixture) was removed, and the precipitate was recovered by vacuum filtration using Whatman paper #1 and labeled CLA-β-CD. The recovered CLA-β-CD was dried in a desiccator under vacuum for 24 h. The control (without NSO-CLA) β-CD microparticles were prepared in tangent. The dried CLA-β-CD microparticles were stored at 4 °C.

To assess the inclusion efficiency, 0.5 g of CLA-β-CD obtained above was stirred into a flask containing 50 mL distilled water and 15 mL hexane following the procedure for encapsulation efficiency [29]. The mixture was ultrasonicated at 60 °C for 5 min and then separated using a separatory funnel. The lower layer was then transferred to another flask and for additional extractions with hexane, undergoing ultrasonication three more times. After pooling the hexane fractions and drying over anhydrous Na_2_SO_4_, the hexane was removed using a rotary evaporator, and the recovered fraction was weighed. The total mass of the lipid fraction recovered was then compared to the initial mass of CLA used in the complexation process to determine the inclusion efficiency.

#### 2.2.5. Oxidative Stability

The oxidative stability of CLA-β-CD was compared with that of NSO-CLA using peroxide value (PV), para-anisidine value (p-AV), thiobarbituric acid (TBA) value and total oxidation value (TOTOX). These indices were selected to characterize different stages of lipid oxidation. PV assay was used to assess the formation of primary oxidation products, particularly lipid hydroperoxides, whereas p-AV assay was used to evaluate secondary oxidation products, particularly aldehydes formed following hydroperoxide decomposition. TBA value was included as an additional measure of secondary lipid oxidation through the determination of malondialdehyde and related TBA-reactive products. TOTOX, calculated from PV and p-AV, was used to provide an overall assessment of oxidative deterioration by integrating both primary and secondary oxidation products [30].

For PV determination, aliquots of CLA-β-CD and NSO-CLA were incubated in a shaking water bath (Amerex Instruments, Concord, CA, USA) at 35 °C and 150 rpm for 96 h. Aliquot were collected every 24 h. PV of the NSO-CLA and CLA-β-CD particles were determined per AOCS Official Method Cd 8-53 for reduced sample weight and expressed as milliequivalents O_2_/kg (mEq O_2_/kg) of oil. p-AV was determined using AOCS Official Method Cd 18-90. To determine the TBA value using the AOCS Official Method Cd 19-90, 100 mg CLA-β-CD or NSO-CLA were dissolved in 25 mL 1-butanol. Aliquots of 5 mL of the resulting mixture was added to 5 mL TBA reagent (2 mg/mL in 1-butanol) in a water bath (Amerex Instruments, Concord, CA, USA) at 95 °C for 2 h. The absorbance of the cooled samples was read at 532 nm in a GENESYS^TM^ 10s UV-VIS spectrophotometer (Thermo Fisher Scientific Co., Fair Lawn, NJ, USA). The total amount of intermediate polar compounds (peroxides and aldehydes) that result from lipid oxidation was used to calculate TOTOX as: TOTOX = (2 × PV) + p-AV.

#### 2.2.6. Characterization

Particle size distribution of the dried β-CD and CLA-β-CD were evaluated by a laser diffraction particle size analyzer (LS 13320, Beckman Coulter, Brea, CA, USA). The obscuration during the measurement amounted to ~10% with a pump speed set at 70%. The morphology of the β-CD and CLA-β-CD was examined using an environmental scanning electron microscope (ESEM) (Quanta FEG 250; FEI, Hillsboro, Oregon, AZ, USA) operated at 3 kV under low-vacuum conditions to minimize the volatilization of embedded compounds. Samples were mounted directly on a sample holder, and images were obtained at magnifications of 500×, 1000×, and 2000×. The variation in crystal structure before (β-CD) and after complexation (CLA-β-CD) was analyzed using a X’Pert Pro PW3040/60 system (PANalytical, Almelo, Netherlands) with Cu-Kα radiation at an accelerated voltage of 30 kV and a current of 10 mA following the method described [19]. All scans were performed at the 1*θ* angle ranging from 2 to 30° with an increment of 0.02. Fourier transform infrared (FTIR) spectroscopy was used to evaluate changes in the molecular environment associated with formation of the CLA-β-CD complex. The analysis was conducted using an Agilent Cary 630 FTIR spectrometer (Agilent, Santa Clara, CA, USA) equipped with a diamond ATR module. FTIR spectra were recorded over the range of 4000 to 400 cm^−1^ at a resolution of 4 cm^−1^, averaging three scans. Air was used as the background for the scans. Samples were gently placed on the diamond crystal, and the pressure arm was lowered to secure them against the crystal surface. Complete FTIR spectra were collected and compared to an open beam background spectrum.

### 2.3. Statistical Analysis

All data analyses were performed in SAS Version 9.4 (SAS Institute Inc., Cary, NC, USA), with the procedures UNIVARIATE and GLM. SAS PROC UNIVARIATE was used to calculate CLA means and standard errors. Combining all the treatment combinations as one factor with 27 levels, one-way analysis of variance (ANOVA) was performed in SAS PROC GLM and was followed by Tukey’s post hoc tests (*p* < 0.05) in the same procedure to differentiate among the treatment combinations, after the main effects had been found highly significant (*p* < 0.0001) with the *F*-test of the one-way ANOVA.

## 3. Results and Discussion

### 3.1. Alkali Isomerization and Crystallization of CLA

The fatty acid profiles of native NSO, isomerized oil, and the purified CLA fraction is shown in Table 1. Native NSO contained 38.25% linoleic acid and 0.034% cis-9, trans-11 CLA. The linoleic acid content was higher than the 27.91% previously reported for NSO by Akonjuen & Aryee [13]. Although both studies used hexane for oil extraction, the extraction conditions differed, particularly the seed:hexane ratio. These differences, together with variation in seed source and analytical conditions, may have contributed to this variation. Following alkali isomerization, the content of cis-9, trans-11 CLA increased to 20.00%, while linoleic acid decreased to 5.00%. This simultaneous decrease in linoleic acid and increase in CLA is consistent with the conversion of linoleic acid during alkaline isomerization, as also demonstrated for sesame oil by Ramírez-Brewer et al. [11]. The content of arachidic acid increased from 5.98% in native NSO to 14.40% in the isomerized fraction, whereas palmitic, stearic, and oleic acids did not differ significantly between the native and isomerized oils. The increase in the relative abundance of arachidic acid may reflect an apparent concentration effect and changes in other fatty acids rather than its synthesis during isomerization [31]. Urea complexation further increased cis-9, trans-11 CLA from 20.00% in the isomerized fraction to 46.42% in the purified fraction, demonstrating enrichment of the quantified CLA isomer following crystallization. Recent work confirms that urea complexation remains useful for fatty acid enrichment and that its efficiency is influenced by molecular structure, urea:fatty acid ratio, solvent composition, crystallization temperature, and other processing conditions [32,33,34]. 

**Figure 1:**
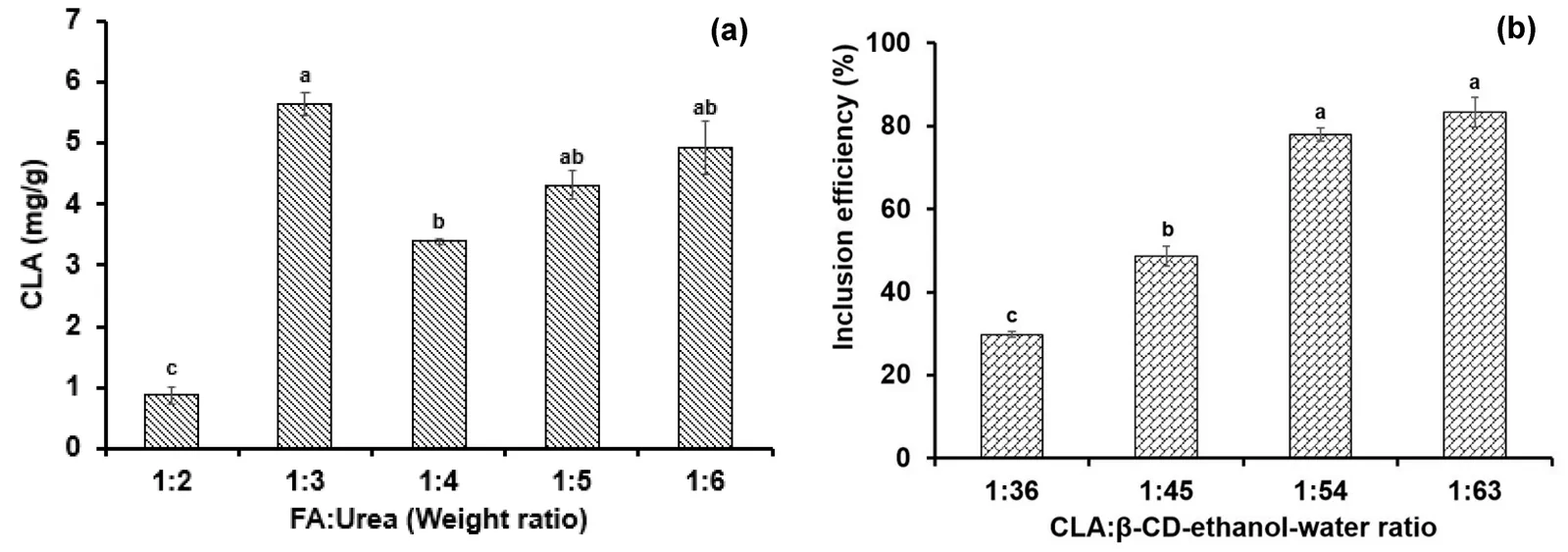
Effect of (**a**) conjugated linoleic acid (CLA) to urea weight ratios on purified CLA content, and (**b**) oil to β-cyclodextrin-ethanol-water ratios on inclusion efficiency.

The effect of fatty acid:urea ratio on CLA enrichment was further evaluated using ratios of 1:2–1:6 (*w*/*w*) (Figure 1a). The highest amount of purified cis-9, trans-11 CLA was obtained at a ratio of 1:3 (*w*/*w*), which was significantly higher (*p* < 0.0001) than the amounts obtained at the other ratios evaluated. The CLA content decreased by ~40% when the ratio was increased from 1:3 to 1:4, followed by a partial increase at 1:5 and 1:6; however, the amounts obtained at 1:5 and 1:6 were not significantly different. The lowest purified CLA content was obtained at a fatty acid:urea ratio of 1:2 (*w*/*w*). The non-linear response across the tested ratios indicates that increasing the urea proportion did not result in a corresponding increase in CLA. The urea:fatty acid ratio is an important determinant of complexation selectivity, and increasing the amount of urea beyond an optimal ratio does not necessarily improve the purity or recovery of the target fatty acid [33,35]. At higher urea ratios, a portion of PUFAs may also become incorporated into the urea complex, thereby reducing their recovery in the desired fraction [33,35]. This interpretation is consistent with urea-complexation studies showing that target fatty acid purity and recovery depend strongly on the urea:fatty acid ratio and that an optimum ratio is generally observed rather than a continuous increase with increasing urea [32,33]. Du et al. [34] also demonstrated that CLA isomer separation by urea complexation depends on differences in molecular configuration and reported selective complexation behavior for cis-9, trans-11 CLA. Thus, the 1:3 ratio represented the most effective condition among the ratios evaluated in the present study.

### 3.2. Product Performance Optimization

The effects of reaction temperature, oil amount, and reaction time on CLA production were evaluated across the 27 treatment combinations of the factorial design (Table 2). The one-way ANOVA showed a significant difference among the treatment combinations (*F* = 18.64, *p* < 0.0001), supporting subsequent multiple comparisons using Tukey’s test (*p* < 0.05). Among the 27 treatment combinations, the highest CLA content (24.99%) was obtained at 180 °C using 2.5 g NSO for 3 h (Table 2). This indicates that the most favorable combination of reaction conditions for NSO was not simply associated with the highest temperature, longest reaction time, or largest quantity of oil, but with a specific combination of these factors. Based on Tukey’s Minimum Significant Difference computed to be 7.27%, the mean CLA content of 24.99% is significantly different from the mean CLA contents of all 26 other treatment combinations. By comparison, the second highest mean CLA content of 17.12% (obtained at 150 °C using 7.5 g NSO for 3 h) is significantly different from the mean CLA contents of only 11 treatment combinations (i.e., those with a mean CLA content of 9.27% and below). Previous studies have also reported different favorable conditions for CLA production depending on the starting oil and reaction system. Shen & Peng [36] reported maximum CLA conversion from peanut oil at 200 °C, 2.6 h, and 7% KOH, whereas Ramírez-Brewer et al. [11] reported the highest conversion from sesame oil using 7% NaOH for 2 h. These differences support the need to determine favorable reaction conditions experimentally for each oil rather than assuming that conditions optimized for one oil will apply directly to another.

### 3.3. Inclusion Efficiency

Figure 1b shows the inclusion efficiency obtained at different ratios of purified CLA to β-CD. Inclusion efficiency increased with the amount of β-CD used, with the highest value (85%) observed at a CLA:β-CD ratio of 1:63 (*w*/*w*). This value was higher than the 64% reported for fish oil loaded in β-CD by Ghorbanzade et al. [20], but lower than the 94.59% reported for garlic oil–β-CD complexes by Li et al. [21]. Kong et al. [19] observed a 94.3% encapsulation efficiency and high recovery yield for tea tree-β-CD microcapsules, attributing these results to the volume fraction of ethanol used. These differences among studies may reflect variations in the guest material, β-CD proportion, solvent composition, and preparation conditions [19,20,21], and therefore direct comparison should be made cautiously.

The amount of CLA-β-CD complex recovered in the present study was lower than the initial amounts of oil and β-CD used, suggesting some material loss during complexation, separation and recovery. Such losses may also be influenced by formulation and solvent conditions. Despite this, the progressive increase in inclusion efficiency with increasing β-CD proportion indicates that greater availability of β-CD favored incorporation of the CLA-rich fraction within the tested range. However, since 1:63 was the highest ratio evaluated, the 85% inclusion efficiency represents the highest value obtained under the conditions tested rather than the maximum achievable efficiency. Ratios above 1:63 were not investigated; therefore, further studies are needed to determine whether additional β-CD would improve inclusion efficiency or whether the system approaches a plateau at higher ratios.

### 3.4. Oxidative Stability

The production of hydroperoxides (primary oxidation products) as measured by the PV assay in NSO-CLA showed an increasing trend during the storage period, ranging from 1.81 at the beginning (0 h) to 2.05 mEq/kg sample at the end of the storage period (96 h) (Table 3). Recent studies on CLA and other PUFA-rich lipids have also demonstrated the formation of primary oxidation products during storage. Li et al. [15] reported reduced formation of primary and secondary oxidation products in CLA emulsions stabilized with glycosylated whey protein hydrolysates, while Ghorbanzade et al. [20] observed lower PV in fish oil complexed with β-CD than in the corresponding non-complexed oil. NSO, rich in PUFAs is also susceptible to oxidative deterioration. Nguyen et al. [37] recently showed the oxidative stability of NSO during heat treatment was influenced by its fatty acid composition and other compositional factors, including phytosterols. Similarly, the PV of CLA-β-CD increased during storage but remained lower than that of NSO-CLA. The relatively higher PV of NSO-CLA may reflect its greater exposure to oxygen and other pro-oxidant conditions, whereas β-CD complexation may have reduced this exposure. This protective effect is consistent with Ghorbanzade et al. [20], who reported reduced peroxide formation following β-CD complexation of fish oil. For comparison with established oil quality specifications, the PVs of both NSO-CLA and CLA-β-CD remained below the maximum PV limit of 10 mEq O_2_/kg set for refined vegetable oils and the 5 mEq O_2_/kg maximum for fish oils throughout the storage period [38,39]. 

The p-AV, which measures secondary oxidation products, increased gradually in both NSO-CLA and CLA-β-CD during the first 48 h of storage (Table 3). Recent work on NSO also demonstrated increasing secondary oxidation during thermal exposure, with Nguyen et al. [37] reporting higher p-AV in PUFA-rich NSO and soybean oil than in oils containing lower proportions of linoleic acid. The higher p-AV observed in NSO-CLA may result from the decomposition of unstable primary oxidation products into aldehydes as lipid oxidation progresses [12]. In contrast, the significantly lower p-AV of CLA-β-CD throughout storage suggests that complexation reduced the formation of secondary oxidation products. Akonjuen & Aryee [13] reported a similar protective effect in NSO, where PV, p-AV, and TBA value were consistently lower in encapsulated NSO than in the free oil throughout 21 days of storage, with the alginate–Bambara groundnut protein isolate providing greater protection than alginate alone. The sharp increase in p-AV after 48 h in both NSO-CLA and CLA-β-CD may indicate increased conversion of accumulated hydroperoxides into secondary oxidation products as storage progressed [12].

TBA values increased continuously in both the free and complexed samples during storage, although the increase was significantly greater in NSO-CLA than in CLA-β-CD (Table 3). TBA value increased by 185.79% in NSO-CLA compared with 56.90% in CLA-β-CD, further indicating greater oxidative protection following β-CD complexation. This finding agrees with Li et al. [15] who reported lower TBA-reactive substances in CLA emulsions stabilized with glycosylated whey protein hydrolysates, further supporting the ability of delivery systems to reduce secondary CLA oxidation.

TOTOX provides an overall indication of oxidative deterioration by combining primary and secondary oxidation values. This approach has also recently been applied to evaluate oxidative changes in NSO during thermal treatment [37]. Recent studies of vegetable oils have delineated TOTOX values under 10 as indicative of fresh and high-quality oil. In contrast values ranging from 10 to 20 suggest the onset of oxidation, whereas values above 20 point to substantial oxidation or rancidity [40]. Between 0 and 96 h, TOTOX increased by 92.18% in NSO-CLA, whereas only a small increase of 32.09% was observed in CLA-β-CD over the same period (Table 3). CLA-β-CD therefore maintained a TOTOX value below 5 after 96 h of storage at 35 °C. Overall, the consistently lower TOTOX of CLA-β-CD compared with NSO-CLA further demonstrate that β-CD complexation reduced the extent of lipid oxidation during storage [13,20]. No exogenous antioxidant was included in the present formulation as the study was designed to assess the protective effect of β-CD complexation on NSO-CLA independently, rather than as a simulation of commercial shelf life. The potential combined effect of β-CD complexation with food-grade antioxidants and beyond 96 h of testing should be evaluated in future studies, particularly for longer-term storage and food applications.

### 3.5. Particle Size Distribution

**Figure 2:**
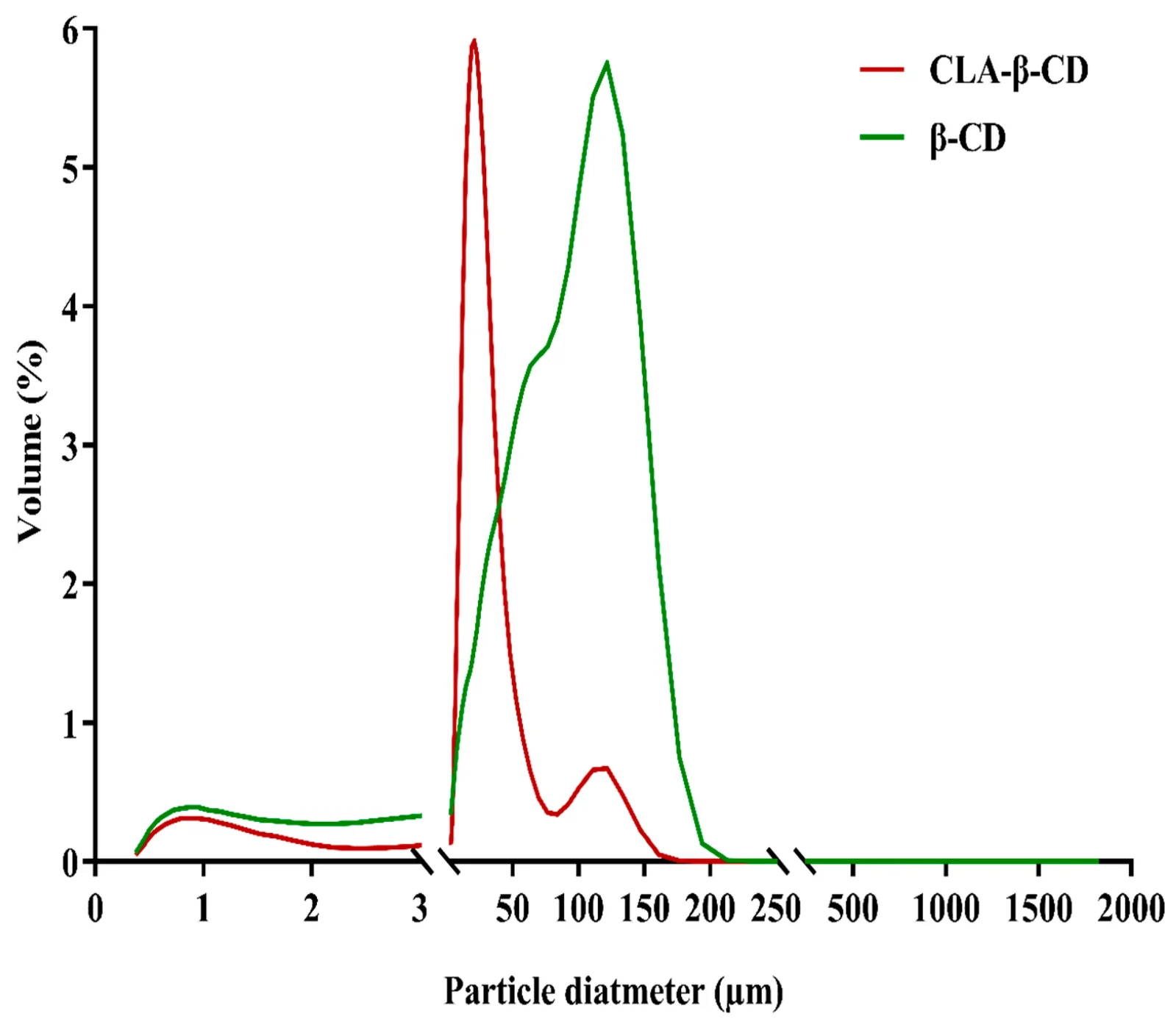
Particle size distribution of β-cyclodextrin (β-CD) and complexed NSO-CLA (CLA-β-CD).

The particle size distribution of β-CD and CLA-β-CD were evaluated using average particle size (D50) and the size at the 90th percentile (D90). The D50 and D90 values represent the particle diameters below 50% and 90% of the cumulative particle size distribution fall, respectively. β-CD had a D50 of 59 µm and a D90 of 139.2 µm, whereas CLA-β-CD had a D50 of 19.73 µm and a D90 of 44.38 µm (Figure 2), indicating a reduction in particle size in the inclusion complex. The reduction in particle size may be associated with changes in β-CD molecular packing, recrystallization, and aggregation during formation of the CLA-β-CD complex rather than a direct size-regulating effect of CLA. Interaction of CLA with β-CD during complexation may alter intermolecular interactions and subsequent crystal growth, resulting in particles with smaller dimensions and different aggregation behavior. This interpretation is supported by the XRD results of the present study, which showed changes in the crystalline organization of β-CD following CLA incorporation. Liu et al. [41] also reported changes in β-CD crystalline packing following oleic acid inclusion, while Kong et al. [19] observed smaller particle sizes after incorporating tea tree oil into β-CD. 

### 3.6. Scanning Electron Microscopy Analysis

**Figure 3:**
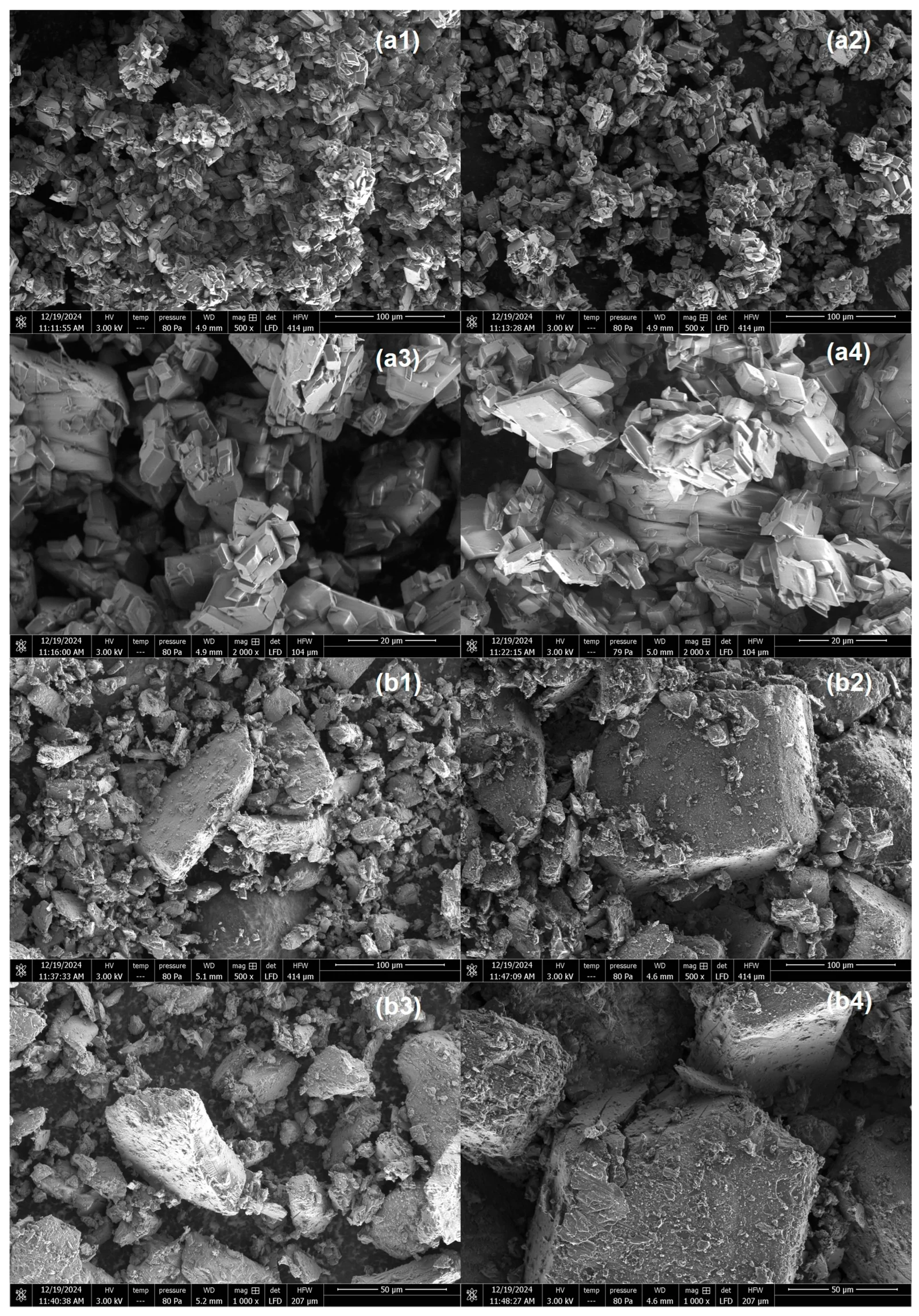
Scanning electron microscope (SEM) micrographs of β-cyclodextrin (β-CD) at 500× (**a1**,**a2**) and 2000× (**a3**,**a4**) magnification and complexed β-cyclodextrin and NSO-CLA (CLA-β-CD) at 500× (**b1**,**b2**) and 1000× (**b3**,**b4**) magnification.

The β-CD exhibited non-uniform, massive or lamellar plate-like structures with fully developed crystals of diverse dimensions (Figure 3(a1, a2)). The crystal surfaces featured distinct minute particles and various cracks or linear markings (Figure 3(a3, a4)). These observations align with the findings reported by Kong et al. [19] who described plain β-CD as irregular massive or lamellar plates with well-grown crystals of varying sizes and surface fractures or striations. Following inclusion complex formation, CLA-β-CD microparticles exhibited irregular, relatively homogeneous block- or plate-like structures (Figure 3(b1–b4)), demonstrating a clear change in morphology compared with the β-CD. Recent studies also show that incorporating lipids into β-CD can change its morphology. Huang et al. [22], reported aggregated structures after forming β-CD inclusion complexes with fatty acid ethyl esters from flaxseed oil, with hydrogen bonding contributing to the interaction between the fatty acids and β-CD. These morphological differences might reflect variations in the chemical composition of the guest materials and the conditions used for forming inclusion complexes, particularly since this present study employed a NSO-CLA lipid fraction.

### 3.7. Fourier Transform Infrared Spectroscopy

**Figure 4:**
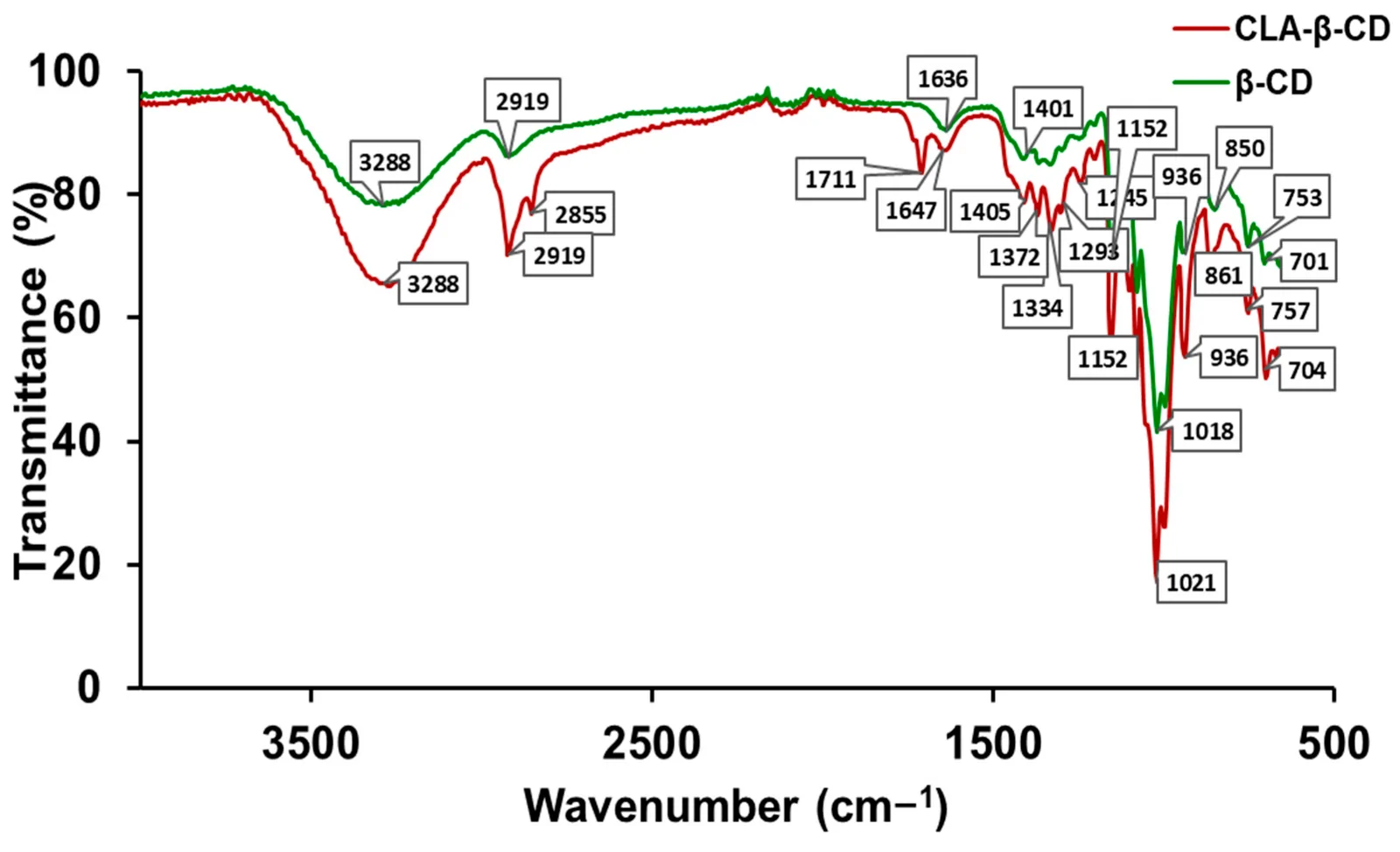
Fourier transform infrared (FTIR) spectra of β-cyclodextrin (β-CD) and complexed NSO-CLA (CLA-β-CD).

FTIR spectroscopy was used to evaluate changes in the molecular structure of β-CD following inclusion complex formation with NSO-CLA (Figure 4). The spectrum of β-CD showed a broad band at 3288 cm^−1^ associated with O–H stretching and a band at 2919 cm^−1^ associated with C–H stretching. These bands were retained at similar positions in CLA-β-CD. Several other β-CD-associated bands were also retained after complexation but showed small shifts, including from 1636 to 1647 cm^−1^, 1401 to 1405 cm^−1^, 1018 to 1021 cm^−1^, 850 to 861 cm^−1^, 753 to 757 cm^−1^, and 701 to 704 cm^−1^. In contrast, the bands at 1152 and 936 cm^−1^ remained at similar wavenumbers. CLA-β-CD also exhibited bands at ~2855 and 1711 cm^−1^ that were not evident in the β-CD spectrum. These bands are associated with CH_2_ stretching in the hydrocarbon chains of fatty acids and C=O stretching of fatty acid carboxyl groups, respectively, supporting the presence of the CLA-rich lipid fraction in the β-CD formulation [41].

Additional bands at 1372, 1334, 1293, and 1245 cm^−1^ were observed in the CLA-β-CD spectrum. These bands occur within the fingerprint region, where C–H bending, O–H deformation, and C–O/C–O–C vibrations may overlap, and therefore cannot be assigned uniquely from the present spectra. Overall, the retention of characteristic β-CD bands, together with the observed shifts and additional lipid-associated bands, indicates changes in the molecular environment of β-CD following incorporation of NSO-CLA. These spectral changes are consistent with non-covalent host–guest interactions during inclusion complex formation rather than the formation of new covalent bonds [18,19]. The retention of β-CD-associated bands after complexation is consistent with Kong et al. [19], who also reported preservation of characteristic β-CD bands in the inclusion complex. Puebla-Duarte et al. [18] also observed shifts in β-CD-associated O–H and C–H stretching modes in the inclusion complex, including shifts from 2920 to 2930 cm^−1^ and from 3279 to 3291 cm^−1^.

### 3.8. X-Ray Diffraction Analysis

**Figure 5:**
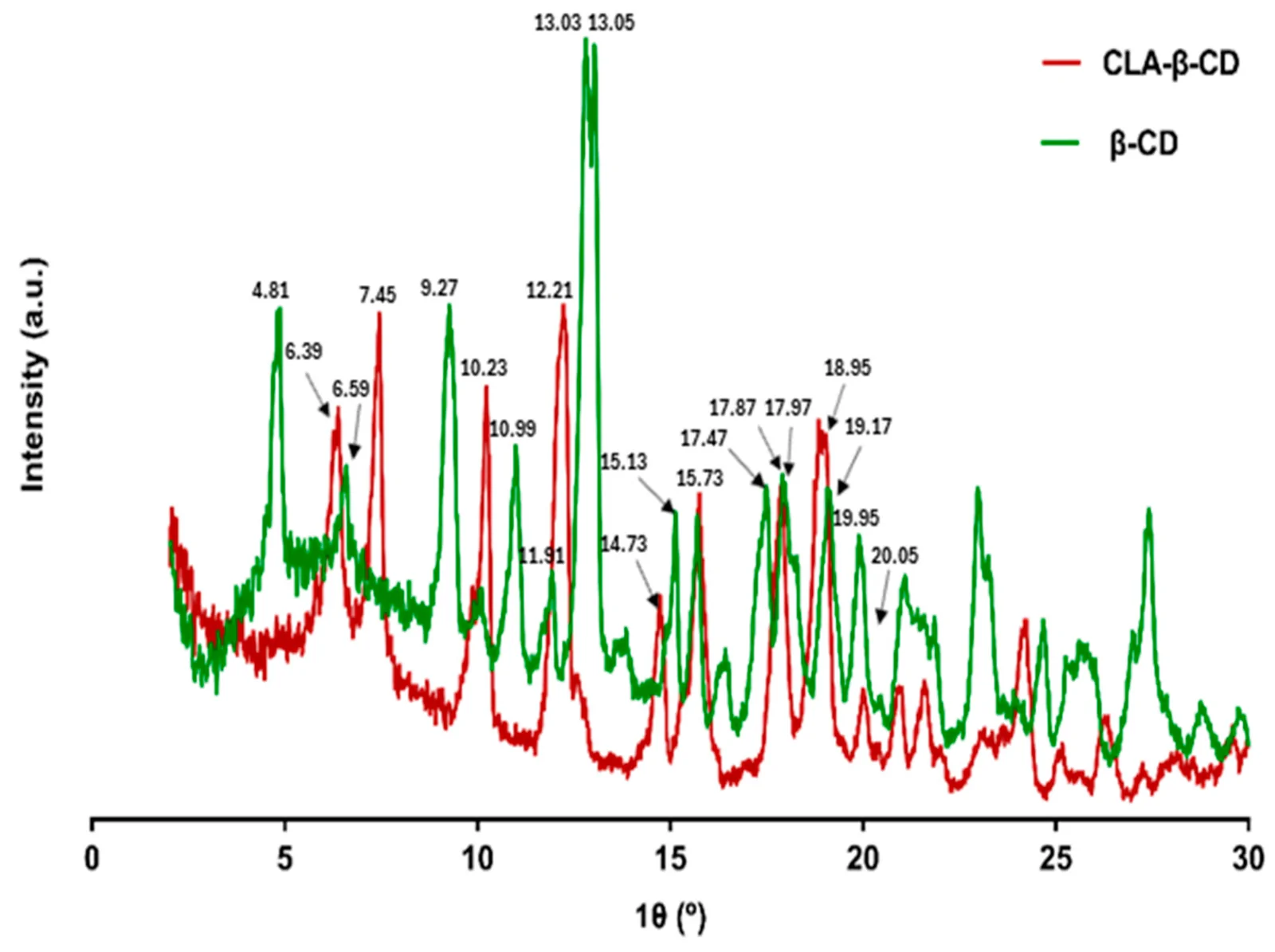
Diffractogram of β-cyclodextrin (β-CD) and complexed NSO-CLA (CLA-β-CD).

XRD analysis was used to evaluate the crystalline structure of β-CD before and after incorporation of NSO-CLA. The XRD pattern of CLA-β-CD microparticles differed from that of β-CD (Figure 5). CLA-β-CD microparticles showed prominent diffraction peaks at 6.39°, 7.45°, 10.23°, and 12.21°, whereas β-CD displayed distinct peaks at 4.81°, 6.59°, 9.27°, 10.99°, 13.03°, and 13.05°. The disappearance of the 4.81° reflection and the appearance of a reflection at 7.45°, together with changes in reflection position and intensity, indicate that incorporation of NSO-CLA altered the crystalline organization of β-CD while preserving its overall crystalline character. β-CD can exhibit two main crystalline packing arrangements, commonly described as cage and channel-type structures. In cage-type packing, adjacent β-CD molecules restrict continuous alignment of the cavities, whereas in channel-type packing, the β-CD molecules are stacked such that their cavities form continuous channels [19,42]. Characteristic reflections of cage-type β-CD have been reported near 5.5°, 6.5°, 9.6°, 11.0°, 12.9°, and 13.5° [43]. The reflections observed for β-CD in the present study at 6.59°, 9.27°, 10.99°, and 13° correspond closely to these reported positions, indicating that the β-CD pattern was predominantly consistent with cage-type packing. Following NSO-CLA incorporation, the diffraction pattern shifted toward reflections characteristic of channel-type β-CD. Hasegawa et al. [44] reported channel-type β-CD reflections at 6.08° (001), 7.27° (1̅10), 10.02° (110), and 12.18° (002). Based on this comparison, the corresponding CLA-β-CD reflections observed at 6.39°, 7.45°, 10.23°, and 12.21° were tentatively indexed as (001), (1̅10), (110), and (002), respectively.

The close agreement between these indexed reflections and the CLA-β-CD pattern provides further evidence that NSO-CLA incorporation promoted reorganization of β-CD from predominantly cage-type packing toward a channel-type arrangement rather than formation of a completely new crystalline phase. This interpretation is consistent with previous studies reporting structural reorganization of β-CD following inclusion of hydrophobic compounds. Kong et al. [19] observed changes in the diffraction pattern of tea tree oil–β-CD microcapsules that were associated with reorganization from cage- to channel-type packing while Liu et al. [41] reported a similar cage-to-channel transition following inclusion of oleic acid in β-CD. Ogata et al. [45] also observed changes in the β-CD diffraction pattern in the inclusion complex, including the appearance of new reflections in cyclamen aldehyde-β-CD co-precipitates. Puebla-Duarte et al. [18] also reported alterations in the diffraction pattern of a β-carotene-β-CD inclusion complex while maintaining its crystallinity. 

## 4. Conclusions

The present study demonstrated the production of CLA from NSO, with the highest CLA content reaching 24.99% under the conditions evaluated. Purification further increased cis-9, trans-11 CLA to 46.42%, highlighting the potential of NSO as a plant-derived substrate for CLA production. Complexation of NSO-CLA with β-CD produced microparticles with distinct particle size, morphology, molecular interactions, and crystalline organization. FTIR spectral changes were consistent with non-covalent interactions between NSO-CLA and β-CD, while XRD suggested reorganization of β-CD crystalline packing following CLA incorporation without loss of overall crystallinity. These findings support the formation of a CLA-β-CD inclusion complex. Complexation also improved the oxidative stability of NSO-CLA, as reflected by consistently lower peroxide, p-anisidine, thiobarbituric acid, and TOTOX values in CLA-β-CD compared with uncomplexed NSO-CLA during storage. These findings demonstrate the potential of β-CD complexation as a strategy for improving the oxidative stability of NSO-CLA and support further evaluation of the complex as an ingredient in oxidation-sensitive food systems. Future studies should investigate residual alkali and potential heat-induced by-products and evaluate lower-temperature approaches for CLA production. Further characterization of the recovered lipid fractions is also needed to determine the complexation behavior of individual fatty acids and CLA isomers. Additionally, the performance of CLA-β-CD should be evaluated in functional food systems and during extended storage, including its potential combination with suitable food-grade antioxidant.

## Figures and Tables

**Table 1:** Fatty acid composition (mean values ± standard errors) of initial oil, mixtures obtained during alkali isomerization of Njangsa seed oil.

Fatty Acid	Native Oil	Isomerized Oil	Purified Fraction
	%		
Palmitic acid (C16:0)	15.78 ± 2.73 ^a^	15.47 ± 1.73 ^a^	16.80 ± 2.72 ^a^
Stearic acid (C18:0)	15.38 ± 3.44 ^b^	15.27 ± 2.28 ^b^	21.85 ± 1.21 ^b^
Oleic acid (C18:1n9c)	20.24 ± 2.05 ^a^	19.32 ± 1.99 ^a^	3.27 ± 0.42 ^b^
Linoleic acid (C18:2n6c)	38.25 ± 5.32 ^a^	5.00 ± 3.87 ^c^	11.60 ± 0.85 ^b^
Linolenic acid (C18:3n9c)	4.34 ± 0.86 ^b^	10.59 ± 1.65 ^a^	0
Arachidonic acid (C20:0)	5.98 ± 0.95 ^b^	14.4 ± 0.11 ^a^	0
cis-9, trans-11 CLA	0.034 ± 0.00 ^c^	20.00 ± 3.08 ^b^	46.42 ± 3.56 ^a^

Different superscript letters in the same row indicate significant (*p* < 0.05) differences between fatty acids.

**Table 2:** Mean value (± standard error) of conjugated linoleic acid (CLA) content (%) for the 27 combinations of experimental levels of the three input variables.

Rank	X1: Reaction Temperature (°C)	X2: Amount of Oil (g)	X3: Reaction Time (h)	Mean CLA Content (%)	Standard Error
1	180	2.5	3	24.99	3.39
2	150	7.5	3	17.12	1.53
3	180	7.5	6	16.97	1.34
4	150	5.0	3	16.25	1.65
5	150	2.5	1	15.58	0.84
6	150	2.5	3	15.22	0.20
7	180	5.0	1	14.45	1.97
8	180	7.5	1	14.43	1.29
9	180	2.5	1	14.26	0.46
10	180	5.0	3	12.97	1.57
11	170	2.5	3	12.88	0.99
12	170	2.5	6	11.67	0.87
13	150	7.5	6	11.35	1.80
14	180	5.0	6	11.03	0.85
15	180	2.5	6	10.59	2.33
16	170	2.5	1	10.29	2.10
17	180	7.5	3	9.27	1.11
18	150	7.5	1	9.06	0.42
19	170	7.5	3	8.96	1.14
20	150	5.0	1	7.71	0.49
21	150	5.0	6	7.24	0.17
22	170	7.5	1	6.48	0.53
23	170	7.5	6	5.87	0.77
24	150	2.5	6	2.59	0.54
25	170	5.0	6	1.12	0.11
26	170	5.0	3	0.71	0.06
27	170	5.0	1	0.49	0.03

**Table 3:** Oxidative stability of free and complexed conjugated linoleic acid.

Time (h)	PV (mEq O_2_/kg)		p-AV		TOTOX		TBA (mg Malonaldehyde/kg Sample)	
	NSO-CLA	CLA-β-CD	NSO-CLA	CLA-β-CD	NSO-CLA	CLA-β-CD	NSO-CLA	CLA-β-CD
0	1.81 ± 0.04 ^a^	1.71 ± 0.12 ^a^	7.00 ± 0.79 ^b^	0.10 ± 0.03 ^c^	10.62 ± 0.71 ^b^	3.49 ± 0.20 ^a^	9.43 ± 0.60 ^d^	7.75 ± 0.68 ^c^
24	1.85 ± 0.14 ^a^	1.79 ± 0.15 ^a^	8.48 ± 1.28 ^b^	0.11 ± 0.00 ^b^	12.19 ± 0.99 ^b^	3.66 ± 0.36 ^b^	12.71 ± 0.33 ^c^	7.93 ± 0.50 ^bc^
48	1.88 ± 0.10 ^a^	1.82 ± 0.15 ^a^	9.60 ± 0.74 ^b^	0.12 ± 0.02 ^b^	13.36 ± 0.55 ^b^	3.77 ± 0.30 ^a^	18.75 ± 0.23 ^b^	8.85 ± 0.96 ^c^
72	2.03 ± 0.51 ^a^	1.84 ± 0.19 ^a^	15.63 ± 2.67 ^ab^	1.20 ± 0.15 ^a^	19.69 ± 1.66 ^a^	4.47 ± 0.51 ^a^	25.43 ± 0.62 ^a^	10.40 ± 0.45 ^b^
96	2.05 ± 0.22 ^a^	1.87 ± 0.20 ^a^	16.31 ± 1.99 ^a^	1.31 ± 0.33 ^a^	20.41 ± 1.55 ^a^	4.61 ± 0.73 ^a^	26.95 ± 2.05 ^a^	12.16 ± 0.59 ^a^

Different superscript letters in the same column indicate significant (*p* < 0.05) differences.

## Data Availability

The original contributions presented in this study are included in the article. Further inquiries can be directed to the corresponding author.
